# Are Virtual Forests Just for Relaxation, or Can They Enhance the Benefits of Therapy?

**DOI:** 10.3390/healthcare13060621

**Published:** 2025-03-13

**Authors:** You Zhi Hu, Max Beggs, Yu Xue, Sinuo Gao, Junyoung Seok, Yawen Xiao, Ziqi Zhou, Yifei Zhou, Alex Mariakakis, Mark Chignell

**Affiliations:** 1 Department of Mechanical and Industrial Engineering, University of Toronto, Toronto, ON M5S 3G8, Canada; youzhi.hu@mail.utoronto.ca (Y.Z.H.); max.beggs@mail.utoronto.ca (M.B.); yfei.zhou@mail.utoronto.ca (Y.Z.); 2 Department of Computer Science, University of Toronto, Toronto, ON M5S 2E4, Canada; yuyuyu.xue@mail.utoronto.ca (Y.X.); junyoung.seok@mail.utoronto.ca (J.S.); grace.xiao@mail.utoronto.ca (Y.X.); mariakakis@cs.toronto.edu (A.M.); 3 Faculty of Information, University of Toronto, Toronto, ON M5S 3G6, Canada; sinuo.gao@mail.utoronto.ca; 4 Department of Electrical & Computer Engineering, University of Toronto, Toronto, ON M5S 3G8, Canada; zq.zhou@mail.utoronto.ca

**Keywords:** virtual reality therapy, virtual nature, forest bathing, mental wellness, mental health, social anxiety

## Abstract

Forest bathing (Shinrin-Yoku in Japanese) is used as an intervention for improving mental health, with VR being used to create virtual forests for relaxation. Background/Objectives: In this research, we added therapeutic intent to a virtual forest with the goal of reducing social anxiety, with and without therapeutic instruction. Methods: Fifty-eight first-year psychology students were randomly assigned to one of three conditions: virtual forest only, therapeutic exercises only, and both combined. Results: All three conditions enhanced restorative effects equally. However, only the therapeutic exercise-only condition showed a tendency to reduce social anxiety. Participants in the combined condition reported more positive experiences and showed better comprehension of therapy content in the virtual forest. Conclusions: While the non-VR approach may offer immediate relaxation and possible anxiety reduction, combining the virtual forest with therapeutic exercises may yield better outcomes for sustained engagement and understanding over multiple therapeutic sessions.

## 1. Introduction

In a world where there is considerable psychological distress, coupled with limited mental health treatment resources, various technologies promise to potentially make mental health treatments more effective and readily accessible. One class of technologies seeks to create relaxed and mindful states that reduce stress and overall symptomology. A second class of technologies focuses on structured interventions that seek to address specific issues (such as motivational interviewing to reduce addictive behavior, and exposure therapy to treat various fears and anxieties). There has been little previous research on how to combine relaxing technologies with therapeutic instruction, and none that we could find on therapeutic instruction within a virtual forest. In this paper, we address this gap by looking at the impact of using therapeutic instructions in a virtual forest, focusing on social anxiety as the target mental health issue.

### 1.1. Social Anxiety

Social anxiety disorder (SAD) is a silent disorder usually masked as shyness or introversion, delaying diagnosis and treatment [1]. Individuals with social anxiety avoid speaking in public and expressing opinions due to a persistent fear of judgment and negative evaluations [2,3]. It is closely associated with early school dropout and subsequent depression risk [4].

SAD is influenced by a combination of genetic, temperamental, personality, and environmental factors, such as childhood experiences and peer interactions, which contribute to its development and persistence [5]. Early adverse experiences, such as childhood abuse, have been shown to dysregulate the HPA axis, leading to heightened cortisol reactivity and an increased susceptibility to social anxiety [6]. Additionally, SAD is often associated with deficits in emotion regulation, where individuals struggle to manage emotional responses to anxiety-inducing situations, further exacerbating the disorder [7,8]. Emotionally provocative stimuli, particularly negative facial expressions (e.g., anger and contempt), were found to elicit heightened neural responses in brain regions linked to emotion processing in socially anxious individuals, including increased activity in the insular cortex (specifically to angry faces), and the anterior cingulate cortex (faces showing disgust) [7,9,10]. These heightened responses may reflect an over-sensitivity to social cues of disapproval, a characteristic feature of SAD.

SAD disproportionately affects young people. It has an early onset (between 13 and 15 years old), often emerging in childhood or adolescence [11,12,13]. Approximately 9–14% of the young adult population in North America have SAD [14,15,16]. University students are also impacted, with approximately 10% exhibiting elevated social anxiety [17]. Notably, a study on 6825 participants aged 16–29 in various regions across Asia, North America, and South America found that over a third met the threshold for SAD [18]. Thus, social anxiety is pervasive, and its reduction would have a beneficial effect on large numbers of people.

### 1.2. Traditional Intervention to Reduce Social Anxiety

Traditional interventions for treating SAD are variants of cognitive behavioral therapy (CBT) and include applied relaxation, social skills training, and cognitive restructuring [19]. Applied relaxation focuses on progressive muscle relaxation, which is easy for individuals to practice during daily activity. Social skills training helps individuals with SAD to learn and practice social skills using techniques such as behavioral rehearsal, corrective feedback, and positive reinforcement. In cognitive restructuring, therapists work collaboratively with patients to identify and then contradict the often inaccurate, distressing, negative thoughts that tend to occur automatically as an effect of SAD.

Mindfulness-based interventions (MBIs) are alternative treatments for SAD [20,21,22,23]. MBIs encompass a range of psychotherapy techniques centered on developing non-judgmental present-moment awareness and engaging in meditative practices. Practicing mindfulness cultivates attention focusing, self-compassion, and attention shifting while reducing safety behaviors and cognitive distortions [21,24].

### 1.3. Access to SAD Treatment

In a study involving people who reported anxiety or depression (N = 1956), researchers found that 23.6% of the participants had been diagnosed with SAD by healthcare professionals, but only 40% of them were receiving pharmacotherapy or psychotherapy [25]. Another study reported that over 80% of individuals with SAD receive no treatment, while those who did seek treatment only sought help after 15 to 20 years of experiencing symptoms [13].

Barriers to seeking treatment include social interaction fears, shame, stigma, limited information, and financial cost [11,26,27]. Additionally, lengthy appointment wait times and sparse service availability impede access [28,29]. Barriers to treatment are negatively correlated with age, indicating that younger individuals perceive more hindrances [27]. In educational settings, children and adolescents with SAD are prone to be the targets of bullying and teasing, while educators may know little about how to recognize SAD and manage the condition [29,30].

Recent advances in digital therapy and virtual treatment for SAD have increased access to treatment [31]. However, among 1154 online mental health applications, just 38 applications were relevant to SAD, with only 5 focused on symptom management through techniques like relaxation and cognitive bias modification [32]. Well-designed applications can provide various forms of training (in areas such as social skills, cognitive restructuring, mindfulness practices, and exposure therapy), allowing people with mild forms of disorders (not requiring diagnosis and clinical treatment) to receive assistance. However, rigorous studies with randomized-control trials and larger samples are needed to demonstrate the validity of applications [31].

### 1.4. Real and Virtual Nature

With advancements in virtual reality (VR), researchers have explored the potential of virtual nature experiences to provide similar therapeutic benefits to real nature experiences, particularly for individuals in urban environments with limited access to natural settings. The biophilia hypothesis suggests an inherent biological connection between humans and nature, with natural environments enhancing emotional well-being [33]. Gaekwad et al. found that exposure to nature improves affective states more than urban environments, increasing positive emotions and decreasing negative ones.

Virtual nature environments can replicate these effects, offering similar benefits for those unable to access real nature. Studies have shown that exposure to virtual nature can reduce stress, anxiety, and negative emotions while fostering positive psychological states such as increased vitality and relaxation [34,35,36]. A systematic review of 21 studies on virtual nature’s psychological effects found consistent emotional recovery, with virtual nature more effective at reducing negative emotions than boosting positive ones [37]. Another study assessed the impact of daily exposure to virtual nature on college students. It found decreased anxious arousal (panic) and anxious apprehension (worry) after 3–4 weeks of exposure [38].

Additionally, physiological benefits—including decreased heart rate, lower cortisol levels, and reduced blood pressure—have been observed, supporting the potential of VR nature therapy as a viable intervention for mental wellness [39,40,41]. Despite growing interest in VR-based nature therapy, a systematic review highlighted the need for more rigorous research, as many existing studies rely on small sample sizes and lack high-quality experimental designs [42], a problem that has continued up to the time of this writing.

Researchers have examined various virtual nature environments such as forests, oceans, and other natural landscapes. A study assessing 16 different nature environments found that the perceived relaxation scores from sunset beaches, ocean waves, and green forests were the highest [43]. However, some participants commented that they felt unsafe due to the openness of the sea.

Shinrin-Yoku, or forest bathing, originated in Japan in the 1980s as a practice of immersing oneself mindfully in nature using multiple senses to promote relaxation and stress reduction [44,45]. Forest bathing involves engaging with the forest atmosphere without structured guidance and has been shown to provide psychological and physiological benefits [44]. Over time, this practice evolved into forest therapy with structured sessions led by trained professionals, incorporating techniques such as guided meditation, walking, and cognitive behavioral therapy (CBT) to enhance well-being [46,47]. Research supports both structured and unstructured approaches to forest therapy, with researchers striving for well-researched mechanisms that facilitate mental and physical health improvements [44,45,46,47].

Researchers have designed immersive virtual forests that allow users to navigate freely and explore interactive elements within the virtual environment [48,49,50]. For instance, Mohamad Yahaya et al. [49] integrated breathing exercises and stretching activities at checkpoints within the virtual forest, as well as two tasks (collecting red balls and taking photographs). They found that higher-quality audio-visual elements contributed to a stronger sense of presence and spatial awareness, thereby improving the user experience. While their study suggested that tasks may be successfully implemented in a virtual forest without reducing their beneficial effects, the tasks were simple, and only eight participants were used in their study.

Adding interactive features to a virtual nature environment may enhance mental well-being. Wang et al. investigated the intervention effects of urban vs. park environments, with interactive activities such as flying a kite, enjoying the idyllic scenery, fishing, and watering plants [51]. The experiment was conducted once a week over four sessions with 195 participants having mild to moderate anxiety and depression. Both groups (urban and park) experienced a reduction in anxiety and depression.

However, the integration of psychotherapy techniques with interactive features into virtual nature applications remains an emerging research area. One study examined an e-mental health VR application that combined Mindfulness-Based Cognitive Therapy (MBCT) with nature-based activities, such as focusing on a sprouting flower [52]. However, due to a small sample size of 30 participants, the study did not yield significant results, and the study did not consider the impact of using therapy in a virtual forest. Cognitive overload from various sensory stimuli (e.g., visual, auditory, etc.), including potential cybersickness, may have hindered participants’ ability to focus on the mindfulness practice, leading to non-significant results. Cognitive load, related to limited working memory capacity, can reduce learning effectiveness when excessive information overwhelms the working memory system [53]. Factors like task complexity, interaction levels, avatar representation, and cybersickness can all influence cognitive load and learning performance in VR [54,55]. Given the mixed results thus far, and the paucity of research targeting the use of therapeutic instruction within virtual forests, further research is needed to see if therapeutic instruction might enhance, or interfere with, the beneficial properties of virtual forests.

In response to this research gap, we developed a VR application aimed at addressing mild social anxiety by integrating evidence-based therapeutic techniques within a virtual forest setting. Users navigated a virtual forest that was intended to be calming while receiving guided therapeutic instructions from a virtual therapist. These instructions incorporated elements of CBT, meditation, and mindfulness practices, equipping users with coping strategies to manage social anxiety. Our application was designed for individuals who might have been reluctant to engage in traditional in-person counseling.

### 1.5. Research Questions and Hypotheses

The following research questions were addressed in our study:

1. Does the use of a virtual forest setting (with or without therapeutic instruction) reduce social anxiety (and improve self-rated restoration effects) to a greater extent than therapeutic instruction provided without a virtual forest?
2. Is therapeutic instruction (either by itself or in a virtual forest setting) more effective at reducing social anxiety (and improving self-rated restoration effects) than virtual forest bathing (without therapeutic instruction)?
3. Does the addition of therapeutic instructions make a virtual forest less attractive (measured in terms of user experience, immersiveness, and sentiment) than it would otherwise be?

## 2. Materials and Methods

### 2.1. Participants

A total of 58 students (18–25 years old; women = 36, men = 22) were recruited from a first-year psychology course at the University of Toronto and participated in this study. The students received course credit for their participation. Participants were randomly assigned to three different conditions without any pre-screening for baseline social anxiety levels or VR familiarity. Due to ethical requirements, participants were not diagnosed with SAD, and all participants had minor or mild social anxiety rather than clinical levels of anxiety. There were three conditions: Condition A (VR forest only), Condition B (combined VR forest and therapy exercises), and Condition Non-VR (non-VR, i.e., therapy exercises only). Participants from Conditions B and Non-VR needed to participate in two sessions to complete all the checkpoints in their condition. Participants in Condition A only needed to participate in one session as they could complete the walk through the forest in 30 min.

Participants in Condition B and the Non-VR condition had the option of completing only the first session or of also completing a second session (with a minimum of five days between sessions to increase the amount of time that any effect of reducing social anxiety might have) for additional course credit (one credit per session). A total of 9 of the 40 students in the two relevant conditions continued their participation in a second session (18–19 years old; women = 5, men = 4; B = 5, Non-VR = 4, see Table 1). The attrition rate for both Condition B and Condition Non-VR is around 0.77. A chi-square test of independence found no significant difference in attrition between Condition B and the Non-VR condition, suggesting attrition was similar across both conditions and that there was no significant attrition bias. Relatively few participants completed the second session because most participants had either completed the experimental course credits that they required after the first session or wanted to try out other experiments provided by different research labs.

### 2.2. Materials and Apparatus

The materials and apparatus used in this experiment consisted of (1) self-reported questionnaires assessing social anxiety, restoration effects, usability, and immersiveness; (2) Meta Oculus Quest 2 VR headsets and virtual forest application; and (3) therapy instructions.

#### 2.2.1. Psychological Measurements

The Social Interaction Anxiety Scale (SIAS) is a self-report 20-item questionnaire rated on a 5-point Likert scale that evaluates the stress experienced when interacting with others [56]. Researchers have reported good test–retest reliability with an intraclass correlation coefficient of 0.92 over a 4-week interval, and internal consistency with Cronbach’s α = 0.94 when applied to a wide range of people, including graduate students, community volunteers, and patients with SAD [56,57].

We also used a 6-item Restoration Outcome Scale (ROS) to assess a more sensitive and temporary state, i.e., restoration experienced after visiting favorite places, which consisted of relaxation, calmness, attention restoration, and clearing thoughts [58,59,60]. ROS is commonly used to assess forest-related experience in many studies, with Cronbach’s α ranging between 0.87 and 0.92 [59,61,62].

#### 2.2.2. VR Usability Measurements

We used the Virtual Reality Neuroscience Questionnaire (VRNQ) to assess the quality of VR software in terms of user experience, in-game assistance, and VR-induced symptoms and effects [63]. The Cronbach’s α values for each domain of VRNQ were 0.89 for user experience, 0.90 for in-game assistance, and 0,89 for VRISE [63]. We removed 3 of the 20 questions in this questionnaire because they were not relevant to our study (the 3 questions asked about non-existent features in our VR forest: two-handed interaction, how things were picked up, and how easy it was to use items), leaving 17 items that assessed user experience in VR.

After completing the questionnaires, participants were asked about their experience in using the virtual forest (“Please describe your overall experience in the virtual forest. What aspects of the virtual environment, interactions, or content, if any, stood out to you or influenced your experience positively or negatively?”).

#### 2.2.3. VR Headset and Software

Participants engaged in the virtual forest using the Meta Oculus Quest 2 VR headset. The virtual forest application was designed and implemented using Unity (2020.3.26f1 version) by a team of developers from the Interactive Media Lab, University of Toronto. Assets, including forest environment (NatureManufacture’s Forest Environment), animals (WDALLGRAPHICS and PROTOFACTOR, INC), and architectural buildings (StarGames studio) were bought from the Unity Asset Store.

**Figure 1:**
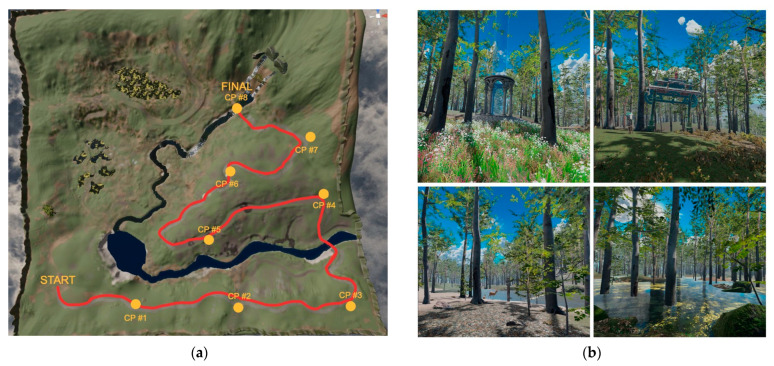
(**a**) Virtual forest map with 8 checkpoints marked in yellow, along with the red path the participants must follow; (**b**) screenshots of the virtual forest.

There were eight checkpoints in the forest, and participants navigated from the start to the destination via a path through the forest (a total of 30 min of walking from the start to the final destination). The path is indicated by a wavy red line on the map shown in the figure, and the checkpoints are represented in the map view as yellow dots (as shown in Figure 1a).

The virtual forest was filled with tall coniferous, and deciduous, trees. The ground was covered with long grass, flowers, and rocks, and occasionally small animals would appear such as deer, squirrels, and foxes, and those animals would move around in the forest. There was also a river with flowing water and the scene portrayed a bright day with partly cloudy skies, adding to the immersive atmosphere of the setting. Tree density varied within the forest, but in general, the sky was quite visible, and the overhead forest canopy was limited (Figure 1b).

In addition to the map that could be accessed at any time with a button press, there were also wooden road signs in the forest pointing the way to the next checkpoint. Each checkpoint had a different architectural building that participants could pause at, or explore, before moving on to the next checkpoint. In Condition B (virtual forest combined with therapy instructions), participants had to stay at the checkpoint area and listen to the audio instructions on techniques to reduce social anxiety (given by the virtual therapist). Links to the video demonstrations of the virtual forest can be found in the Supplementary Materials section.

#### 2.2.4. Experimental Conditions

The following conditions were used in the experiment.

- Condition A—VR forest only: In this VR condition, participants had to navigate through a virtual forest using controllers and joysticks without receiving any therapeutic exercises.
- Condition B—combined VR forest and therapy exercises: Similar to Condition A, participants in this VR condition still navigated through the same virtual forest, but they had to pause their virtual walk and listen to therapeutic exercise instructions that were designed to reduce social anxiety at each checkpoint.
- Condition Non-VR—therapy exercises only: In this Non-VR condition, participants received therapeutic exercise instructions displayed on a laptop, without exploring or walking in the virtual forest. The exercise instructions, identical in content and design to Condition B, were shown on a lab-developed website, accompanied by the same forest sounds used in Conditions A and B. Before the session started, participants were asked to imagine that they were in a forest.

#### 2.2.5. Therapy Instructions

As mentioned in Section 2.2.4, participants from Conditions B and Non-VR had to listen to the virtual therapist’s instructions at each checkpoint. The therapeutic instruction included psychoeducation, meditation exercises, and quizzes that assessed participants’ learning [64]. Materials, from the social anxiety treatment manuals developed by Andrews [65], mindfulness-based stress reduction by Kabat-Zinn [66], and mindfulness-based cognitive therapy developed by Segal et al. [67], were incorporated and modified into our therapeutic instructions on reducing social anxiety and enhancing overall mental wellness. The complete psychotherapy instruction script can be found in Appendix A.

An overview of the 8 checkpoints’ instruction content is provided below:

- Checkpoint 1: In accordance with a typical cognitive behavior therapy first session’s content, this checkpoint focused on psychoeducation [65]. The psychoeducation content was developed based on Andrews’ treatment manual (Pages 3–18). The psychoeducation part was followed by a quick abdominal breathing exercise (from Andrews’ manual) teaching people how to slow down the rate of respiration to cope with shallow and rapid breathing when anxiety level is high.
- Checkpoint 2: This checkpoint introduced the concept of mindfulness, highlighting the concept of focusing on the present moment and becoming aware of thoughts and feelings based on Segal et al.’s mindfulness-based cognitive therapy manual [67]. To help cultivate awareness, participants were asked to engage in the four-senses mindfulness exercise (the five-sense exercise minus taste) to bring awareness to each sense [68].
- Checkpoint 3: We used both Andrews and Segal et al.’s manuals to develop this section of cognitive restructuring [65]. Challenging thoughts and cognitive restructuring are fundamental components of CBT. Participants also received two hypothetical situations, asking the participant to become aware of the thoughts that those situations evoke, to evaluate whether those thoughts are reasonable assessments, and to reappraise the situation if the thoughts were not helpful.
- Checkpoint 4: The fourth checkpoint exercise was the mountain mediation developed by Kabat-Zinn [69].
- Checkpoint 5: Using Andrews’ manual (Pages 33–34), the material in this checkpoint discussed avoidance and its negative impact on social anxiety [65]. While avoidance might seem helpful, it often exacerbates anxiety and prevents personal growth.
- Checkpoint 6: The sixth checkpoint exercise was sound meditation, adapted from Segal et al. to engage participants with forest sound played in the VR headset [67].
- Checkpoint 7: The seventh checkpoint exercise was about self-compassion, which incorporates Hofmann et al.’s Loving-kindness Meditation [70]. It discusses the inevitability of setbacks in conquering social anxiety and emphasizes the importance of treating oneself kindly when encountering those difficult times.
- Checkpoint 8: The eighth checkpoint was similar to a closure session, which guides participants to reflect on what they have learned and how they can apply it in the real world, followed by a simple breathing meditation, Three Minute Breathing Space [66,67].

### 2.3. Procedure

The study was posted on the first-year psychology course’s participant pool system at the University of Toronto in the winter 2024 semester. Eligible participants for enrolment had to be 18 years old or above and not clinically diagnosed with social anxiety disorder. The experiments were carried out in person at the Interactive Media Lab, University of Toronto.

Participants used the provided laptops to complete the consent form, fill in demographic information, and answer a set of questionnaires assessing social anxiety levels and restoration effects. Participants were randomly assigned to one of the three conditions. After watching a three-minute tutorial video on how to complete the VR/Non-VR task, participants were provided with either the Meta Oculus Quest 2 VR headset or the laptop displaying the Non-VR website to start the task. There were two rounds of VR tasks, with a 5 min break between them. Each round lasted about 15 min. Participants were also asked to rate their motion sickness and discomfort levels during the break. After completing the second round, participants were asked to complete another set of questionnaires on user experience, social anxiety levels, and restoration effects as the final step of the experiment. As many participants might not be able to complete all 8 checkpoints in the first session, participants could decide whether they wanted to participate in the second session to complete the whole program. The total time to finish one session was approximately 50 min.

In the second session, the SIAS was administered at the end of the session so that social anxiety at that point could be compared with the baseline social anxiety prior to the first experimental session.

### 2.4. Data Analysis

Data analyses were carried out using R (Version 4.2.1), R Studio (Version 2023.06.0+421), and Python (Version 3.12) to evaluate the effects of different experimental conditions on participants’ social anxiety, quiz performance, user experience, and restoration outcomes.

We began by conducting a principal factor analysis on the Social Interaction Anxiety Scale (SIAS) with Varimax rotation. We chose the number of factors to select based on a scree plot. Item reliability analysis was then used to assess the internal consistency of the selected factor, calculating Cronbach’s alpha for both pre- and post-session data. We then iteratively removed items that did not contribute significantly to the overall reliability, retaining a set of items that had high internal consistency. The average of the SIAS factor items was calculated to develop a scale rating consistent with the summated rating scale method described by Spector [71]. The differences in the resulting social anxiety scale ratings, between pre- and post-session measures, were then analyzed for each condition. This analysis was visualized using Cumming plots, highlighting changes in social anxiety across the three conditions.

In addition to analyzing social anxiety, we conducted a Fisher’s exact test to evaluate differences in quiz accuracies across two conditions (B and Non-VR) in three checkpoints to further assess participant’s understanding of the therapy instructions. User experience was assessed using one-way ANOVA and post hoc Tukey tests, comparing overall quality and enjoyment ratings (from the Virtual Reality Neuroscience Questionnaire (VRNQ)) across conditions. To examine restoration outcomes, a mixed ANOVA was conducted to assess the effects of conditions and phases on restoration ratings (calculated as the average of the six items from the Restoration Outcome Scale).

Furthermore, we analyzed participants’ open-ended comments using NLTK’s Sentiment Intensity Analyzer to compute sentiment scores, which ranged from −1 (most negative) to +1 (most positive). These sentiment scores were then evaluated using one-way ANOVA and independent t-tests to explore differences in sentiment across conditions.

## 3. Results

### 3.1. Social Anxiety Measurement

#### 3.1.1. Factor and Reliability Analysis

**Figure 2:**
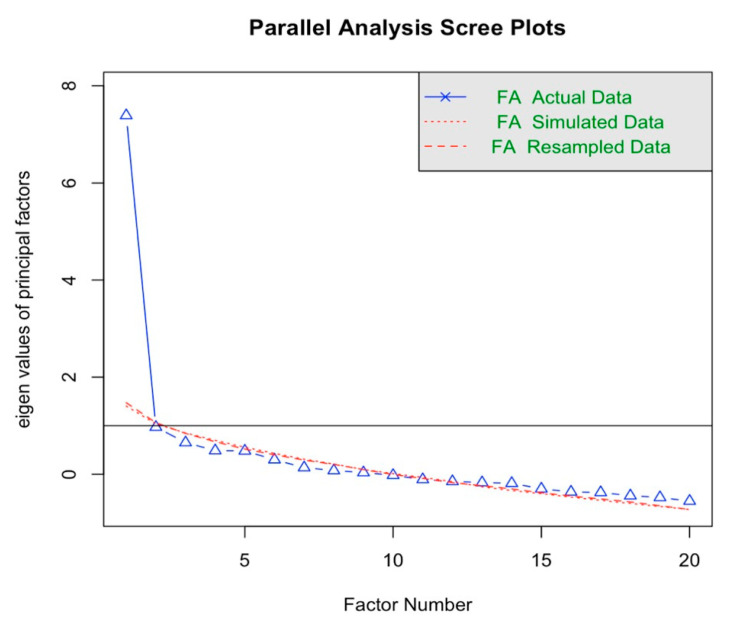
Screen plot: factor analysis on the 20 SIAS items.

We conducted a factor analysis to identify the underlying constructs for the 20-item Social Interaction Anxiety Scale (SIAS). The KMO measure was 0.82, indicating sampling adequacy. Bartlett’s test of sphericity was significant, χ^2^(190) = 569.63, *p* < 0.001, suggesting the factorability of the correlation matrix. The scree plot suggested only one factor (Figure 2). Varimax rotation was applied to achieve a clearer structure. Table 2 shows the factor loadings of the 20 items, in which Item 14, “I have difficulty talking to people I am attracted to”, had the lowest loading with 0.184, suggesting a poor contribution to the factor. All the other loadings were above 0.3.

Item reliability analysis was conducted to identify a parsimonious representation of the social anxiety factor while maintaining strong internal consistency. We began with all 20 SIAS items using post-session 1 data. Items were sequentially removed based on their impact on internal consistency, retaining only those that did not lead to a large drop in Cronbach’s alpha. This led to the inclusion of five items (7, 12, 15, 17, and 19). This five-item scale demonstrated strong internal consistency, with Cronbach’s alpha values of 0.87 for the pre-session data and 0.93 for the post-session data (Table 3).

Peters et al. developed a short form of Social Interaction Anxiety with six items [72]. We also applied Peters’ six items and calculated the Cronbach’s alpha for our pre- and post-session data. The internal reliability for the six-item Peters scale was 0.76 for the pre-session data and 0.81 for the post-session data, both of which were well below the Cronbach’s alpha measures of internal consistency that we obtained for our five-item scale. (0.87 and 0.93), in the pre- and post-sessions, respectively. Thus, we chose to use our five-item version of the SIAS as the short version of the SIAS rather than the Peters scale.

#### 3.1.2. Social Anxiety Change Across Three Conditions

**Figure 3:**
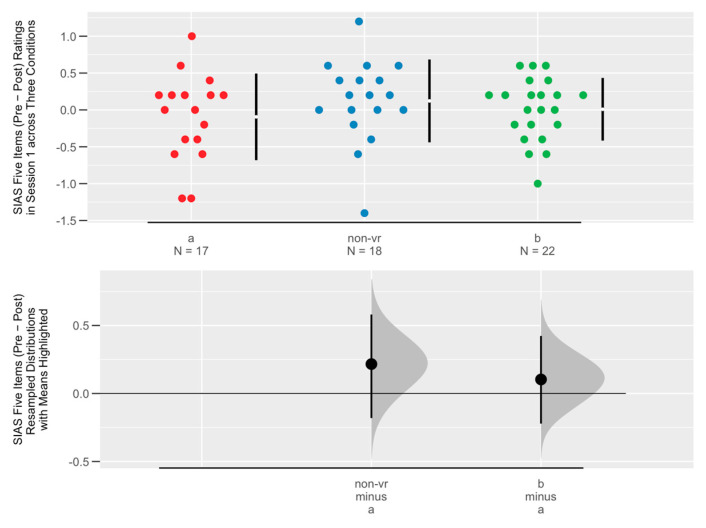
Cumming plot on social anxiety change (pre-post) across three conditions (a (red): virtual forest only as control condition; non-vr (blue): therapy only; and b (green): therapy and virtual forest combined) in Session 1.

We derived a social anxiety scale by calculating the average of the five items from Section 3.1.1. We then subtracted the post-session social anxiety from the pre-session social anxiety, with the resulting differences in social anxiety across the three conditions in Session 1 shown in the Cumming plot in Figure 3. Condition Non-VR (therapy only) showed a greater (but statistically non-significant) drop in social anxiety than conditions A (VR forest Only) and B (combined VR forest and therapy), suggesting therapy-only instructions reduced social anxiety more, possibly because participants in that condition were not distracted by the VR environment and focused more on the therapy content. The virtual forest may make people feel relaxed, but therapeutic instruction may be needed to reduce social anxiety.

**Figure 4:**
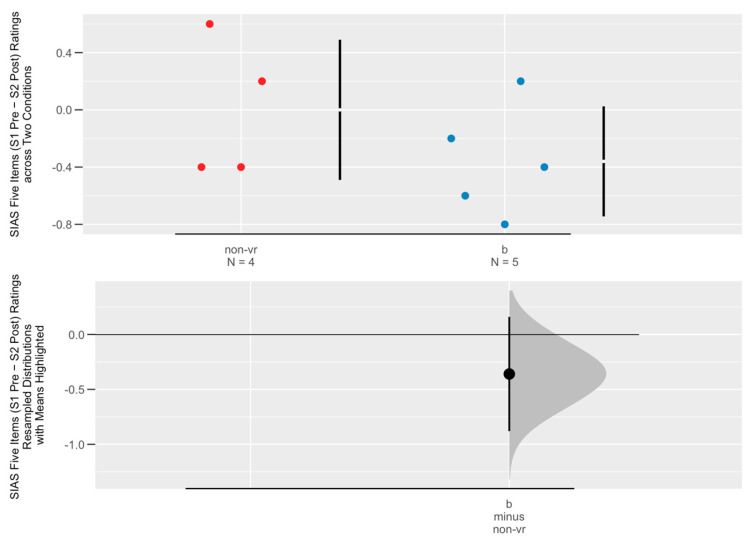
Cumming plot on social anxiety change (S1 pre-S2 post) across two conditions (non-vr (red): therapy only as control condition; b (blue): therapy and virtual forest combined).

The nine participants (five in the combined condition and four in the Non-VR condition) who stayed in both sessions (only participants in Conditions B and Non-VR returned for the second session) were too few for statistical analysis. However, the corresponding Cumming plot in Figure 4 again shows a tendency for Non-VR (i.e., therapy only) participants to report a greater drop in social anxiety (vs. the combined condition).

### 3.2. Checkpoint Quiz Performance

We applied Fisher’s exact test to analyze participants’ performance between Condition B and the Non-VR condition in the first session’s three checkpoints (see Table 4). The results did not differ between the two conditions in the first two checkpoints (Checkpoint 1: *p* = 1; Checkpoint 2: *p* = 1). However, the result indicated a significant difference in Checkpoint 3’s accuracy between the two conditions, *p* < 0.001, odds ratio = 0.04, 95% CI [0.01, 0.18], and Cramér’s V = 0.58. More participants in Condition B answered correctly compared to those in Condition Non-VR, suggesting Condition B helped participants sustain a more accurate understanding of the therapy content over time.

### 3.3. User Experience Questionnaire

**Figure 5:**
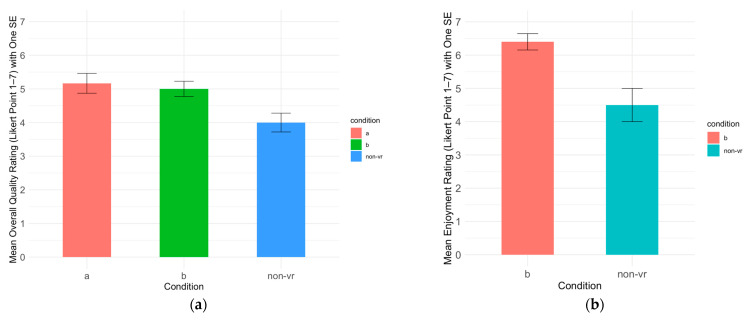
(**a**) Mean overall quality ratings in Session 1 across three conditions; (**b**) mean enjoyment ratings in Session 2 across two conditions.

The participants in the VR conditions assigned higher ratings on user experience, suggesting that VR conditions were preferred to the Non-VR condition. For the Session 1 data, one-way ANOVA showed a significant effect of condition on the user experience overall quality rating, F(2, 55) = 5.40, *p* < 0.01, η^2^ = 0.16. Post hoc Tukey tests revealed that Condition Non-VR (M = 4.00, SD = 1.19) had significantly lower overall quality ratings compared to Conditions A (M = 5.17, SD = 1.25, *p* < 0.05, Cohen’s d = 0.96, 95% CI [0.24, 1.67]) and B (M = 5.00, SD = 1.07, *p* < 0.05, Cohen’s d = 0.89, 95% CI [0.22, 1.56]), as shown in Figure 5a. No differences between the three conditions were found for sound, graphics, immersion, and enjoyment ratings in Session 1.

While only a few participants continued to Session 2, one-way ANOVA revealed a significant effect of condition on enjoyment for those that did, F(1, 7) = 13.37, *p* < 0.01, η^2^ = 0.66. Post hoc Tukey tests revealed that Condition Non-VR (M = 4.50, SD = 1.00, N = 4) had significantly lower enjoyment ratings compared to Condition B (M = 6.40, SD = 0.55, N = 5), *p* < 0.01, Cohen’s d = 2.45, 95% CI [0.36, 4.55], as shown in Figure 5b. No differences between the two conditions were found for sound, graphics, immersion, and overall quality ratings in Session 2. Thus, even though Condition Non-VR showed the greatest reduction in social anxiety in Session 1, we hypothesize that the greater user experience of the two VR conditions in Session 1, and the greater enjoyment of the two VR conditions in Session 2, suggest that VR may help to sustain engagement over multiple sessions.

### 3.4. Open-Ended Comments

**Figure 6:**
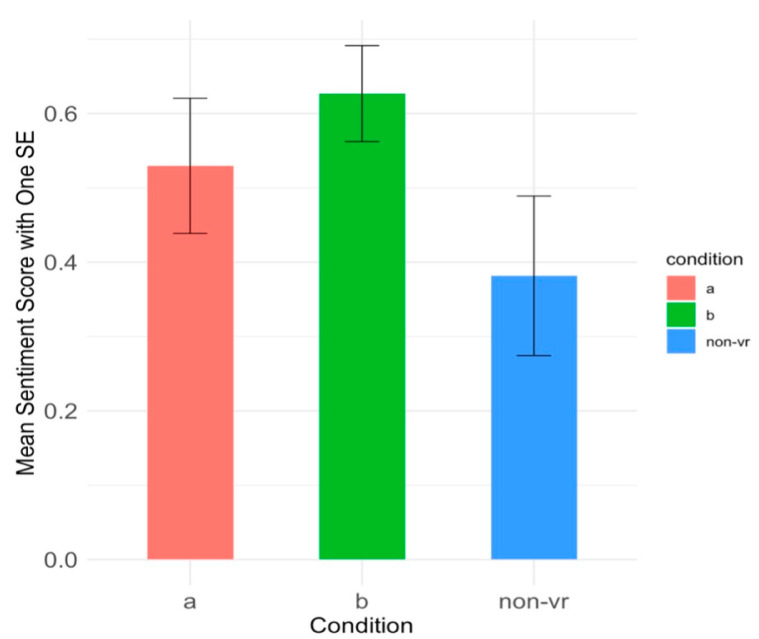
Bar plot of the mean sentiment score with one standard error on participants’ comments across three conditions in Session 1. Condition A: M = 0.53, SD = 0.39. Condition B: M = 0.63, SD = 0.30. Condition Non-VR: M = 0.38, SD = 0.46.

We were interested in whether the presence of VR had an impact on sentiment. Would people prefer to be in the VR forest? We ran an independent samples t-test to examine the effect of VR vs. Non-VR groups on the sentiment score of participants’ comments in the first session. There was a borderline significant difference in ratings between the Non-VR (M = 0.38, SD = 0.46) and the VR (M = 0.58, SD = 0.34) conditions, t(56) = −1.87, *p* = 0.067, 95% CI [−0.42, 0.01], Cohen’s d = 0.53, 95% CI [−1.09, 0.04]. This suggested a greater positivity in the two VR conditions versus the Non-VR (therapy only) Condition. Participants may have perceived the experience as more enjoyable, engaging, or rewarding compared to Condition Non-VR. As shown in Figure 6 and Table 5, participants gave slightly more positive sentiment scores in the VR conditions compared to the Non-VR condition.

**Figure 7:**
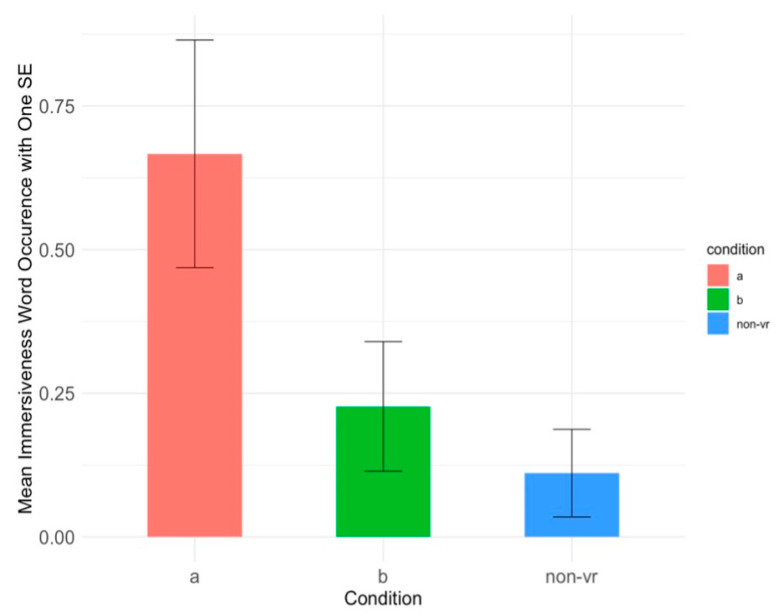
Bar plot of the mean immersiveness word occurrence (with one standard error bars) on participants’ comments across three conditions in Session 1.

We also counted the word occurrence on all the words related to immersiveness (either expressed in single words or in phrases), such as ‘immersive,’ ‘realistic,’ and ‘made me feel like I am actually in the forest’ (see Appendix B for complete word list) from each comment. One-way ANOVA revealed significant differences in word occurrences across the conditions in Session 1, F(2, 55) = 4.39, *p* < 0.05, η^2^ = 0.14 (see Figure 7). Post hoc Tukey tests revealed that Condition A (M = 0.67, SD = 0.84) had significantly higher immersiveness wording than Condition Non-VR (M = 0.11, SD = 0.32), *p* < 0.05, Cohen’s d = 0.87, 95% CI [0.16, 1.58]. Participants in Condition A (virtual forest only) reported a greater sense of immersiveness compared to Condition Non-VR condition with therapeutic instructions only. Condition B, which combined the forest with therapeutic instructions, showed no significant difference from either Condition A or Non-VR, as it fell between the two conditions. The therapeutic instructions in Condition B may have distracted participants from fully engaging with the immersive elements, resulting in lower immersiveness than Condition A but still higher than the Non-VR condition, where no virtual environment was present.

### 3.5. Restoration Outcome Scale

**Figure 8:**
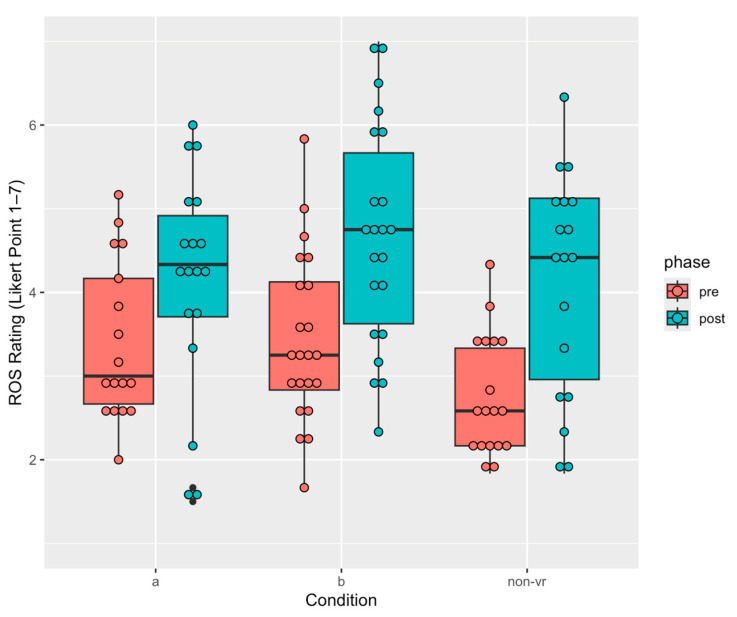
Boxplot of ROS rating across pre- (red) and post- (blue) phases for 3 conditions in Session 1.

We applied mixed ANOVA to examine the effects of conditions and phases on restoration rating in Session 1. There was a significant main effect of phases, F(1, 109) = 27.81, *p* < 0.0001, η^2^ = 0.20 (see Figure 8). Participants from all three conditions showed a significant increase in restoration rating from pre-experiment (M = 3.21, SD = 0.94) to post-experiment (M = 4.34, SD = 1.32), Cohen’s d = 0.98, 95% CI [0.59, 1.38]. This suggested both the virtual forest shown in the two VR conditions, as well as the forest sounds and forest picture in the background in Condition Non-VR, can invoke similar levels of increased restoration.

## 4. Discussion

### 4.1. Non-VR (Therapy Only) Condition Shows Potential for Reducing Social Anxiety

Only the Non-VR therapy showed a tendency to reduce social anxiety levels. Social anxiety is often deeply ingrained in individuals, and it typically requires multiple sessions over a longer period rather than the single-session intervention that most participants underwent in this study. Thus, it is not surprising that statistically significant reductions in social anxiety were not found. In contrast, scores on the ROS increased significantly after the intervention for all three experimental conditions. Consistent with the finding for social anxiety, the increase in ROS from pre- to post- tended to be greater in Condition Non-VR (see Figure 8 in Section 3.5.). A similar trend was found in a real-world setting by Korcz et al. [73], where informal education in a forest setting contributed to greater psychological restoration compared to merely walking in the forest without educational guidance. Their study highlighted the importance of incorporating structured educational tools into forest exposure to enhance psychological well-being.

Condition Non-VR might have produced better results because participants could focus on the therapy instructions without the distractions present in the VR environments (where participants could explore the forest environment). The virtual forest contained many features designed to increase engagement and the quality of the user experience (such as the checkpoint building, animals, forest sounds, and various plants and trees), but this may have also distracted participants from attending fully to the therapeutic instructions.

### 4.2. VR Therapy Enhances Long-Term Engagement and Learning of Therapeutic Information

The quiz performance data revealed that participants in Condition B obtained higher accuracy in Checkpoint 3 (assessing the comprehension of the therapy content) relative to those in Condition Non-VR (see Section 3.2.). Checkpoint 3 occurred after roughly 15 min of interaction task.

On the other hand, sentiment analysis of text comments made after the session showed that sentiment tended to be more positive in the VR conditions vs. therapy only (*p* = 0.067). While this effect was borderline significant, the trend suggests that participants might have felt more positively toward the virtual forest experience, which could have important implications for long-term engagement and treatment adherence. Participants in the VR conditions may have perceived the session as more enjoyable or engaging, potentially increasing their willingness to participate in future therapy sessions. Since treatment adherence is a major challenge in anxiety interventions, virtual forest creating a positive emotional experience in therapy settings could be an important factor in sustaining engagement over time.

Our examination of immersiveness also showed that immersiveness (as indicated by the occurrence of words related to immersiveness in participant comments) was lower in the therapy-only condition. Performance in Checkpoint 3 may have suffered in the therapy-only condition because the lack of immersion and engagement may have made the task more boring. Thus, combining therapy with a virtual forest environment may facilitate more accurate comprehension of therapy content over time, in spite of any distracting effects of the virtual forest.

These findings align with prior research suggesting that immersive virtual nature, when combined with mindfulness practices, can enhance task performance over time. Ch et al. examined the effects of virtual nature combined with audio-guided mindfulness exercises (e.g., breathing and awareness exercises) on remote workers’ creativity over a nine-week period. Their study found that participants demonstrated improved performance on convergent thinking tasks, including a higher number of correct answers, faster response times, and better focus [74]. Similarly, prior research has shown that virtual reality environments can effectively stimulate learners’ interest and enhance engagement [75,76].

There was a tendency towards more reduction in social anxiety in Condition Non-VR. However, the better quiz performance, and ratings on two of the user experience items, in Condition B suggest that VR combined with therapy may offer long-term benefits for therapeutic intervention. The immersive and interactive forest elements may help to sustain engagement and promote deeper comprehension of therapy content over successive therapeutic sessions.

Thus, while Condition Non-VR may tend to reduce social anxiety in a single session, VR conditions might provide better outcomes for long-term learning and engagement, leading to stronger therapeutic benefits overall. An effective VR therapy application would need to optimize the balance between the therapeutic content and immersive forest environment to maintain users’ interest, reduce boredom, and promote sustained learning with an accompanying reduction in social anxiety. This balance is crucial, as overly immersive environments can sometimes lead to cognitive and sensory overload, potentially hindering therapeutic effectiveness [77,78]. We recommend that future research focus on integrating elements that combine the benefits of both approaches, perhaps by incorporating periods of focused therapy content followed by immersive, engaging activities to optimize both relaxation and learning outcomes.

### 4.3. Forest Sound Played a Significant Role in Enhancing Restoration

Self-rated ROS increased after all three conditions in the study as reported in the mixed ANOVA reported in Section 3.5. All three conditions utilized the same background audio, which were forest sounds, including birds chirping, a river flowing, and wind rustling. Many participants commented that the forest sound made the experience more vivid and calming (see Table 6). This aligns with past research showing that exposure to forest sounds can reduce negative emotions and promote relaxation [79,80]. Moreover, Annerstedt et al. emphasize that auditory stimuli play a crucial role in enhancing the recovery aspect of VR-based nature experiences, demonstrating that physiological stress recovery is significantly improved when visual and auditory nature cues are combined. Their findings reinforce the idea that natural soundscapes are not merely supplementary but essential in fostering an effective immersive therapeutic environment [81].

Participants in Condition Non-VR were instructed to imagine being immersed in a forest while viewing a screenshot of the virtual forest as the interface’s background image. The combination of forest sounds and the imagined forest environment produced a restoration effect comparable to that observed in the VR conditions. In Table 6, one participant from Condition Non-VR used words like vivid and immersive. This suggests that the cognitive process of imagining an immersive natural environment, when paired with concordant auditory stimuli, can be as effective in promoting restoration in simulated or imagined virtual experiences. Prior research has similarly shown that guided imagery combined with nature sounds can evoke relaxation responses, reduce anxiety, and manage pain [82,83]. The equivalence in restorative effects between the VR and Non-VR conditions suggests that a cheaper and simpler Non-VR implementation could provide similar benefits in making therapeutic interventions more engaging through guided imagery and natural soundscapes.

## 5. Limitations and Future Research

While our study provides insights into the usability and effectiveness of combining a virtual forest with therapeutic exercises addressing social anxiety in young adults, there were several limitations. One major limitation was our sample size and the high dropout rate. Although the study initially included fifty-eight first-year psychology students with approximately twenty participants per condition, only nine participants remained for the second session. The high dropout rate limits our ability to draw robust conclusions about the longitudinal changes in the effectiveness and usability of our interventions.

Additionally, the duration and frequency of sessions were limited. Social anxiety is a deeply ingrained trait, and reductions in self-rated anxiety within a single session or even over two sessions across two weeks should probably not be expected in most participants. Typically, therapeutic interventions for social anxiety require about 14–20 weekly sessions to observe meaningful changes [84,85]. The short duration of our intervention, with a maximum of two sessions, limits the generalizability of our findings, as social anxiety therapy typically requires a more extended intervention period. Moreover, our study primarily focused on short-term effects, without assessing the long-term impact of VR therapy on social anxiety reduction. Our study’s short duration and limited number of sessions likely contributed to the minimal observed effects on social anxiety. Future research should implement extended intervention periods and follow-up assessments to examine the sustained effectiveness of virtual forest therapy over time.

The demographics of our participants may not be representative of the broader population of young adults, as the study mainly involved first-year psychology students. A more diverse sample, including individuals from various educational backgrounds and a wider age range (18–30 years), would enhance the generalizability of our findings. Furthermore, none of the participants were clinically diagnosed with severe social anxiety (a requirement of our research ethics protocol), and the relatively low initial social anxiety scores made it more challenging to detect significant reductions in anxiety levels post-intervention. Future research should include participants with varying levels of social anxiety severity and pre-screen for the level of social anxiety to balance it across conditions and provide a more sensitive measure of intervention effectiveness.

We derived a 5-item scale extracted from the Social Interaction Anxiety Scale (SIAS), which showed a strong correlation with the original 20-item scale (r > 0.9, *p* < 0.05), suggesting that a concise and less unwieldy version of the SIAS should capture most of the relevant information. Peters et al. developed a shortened six-item version of the SIAS, which was also applied in our analysis [72]. We calculated the Cronbach’s alpha for the six-item Peters scale and found internal reliability scores of 0.76 for the pre-session data and 0.81 for the post-session data, both of which were lower than the reliability measures we obtained for our custom five-item scale (0.87 and 0.93 for pre- and post-session data, respectively). Based on these findings, we chose to use our five-item version of the SIAS rather than the Peters scale. However, the variability in the reliability of the two shortened scales in our study suggests a need for further research to see whether either of these shortened versions, or others yet to be developed, can demonstrate stability across different samples. A short version of the SIAS should be particularly useful in settings where researchers want to use a range of instruments, and time and participant fatigue may become concerns.

More broadly, new measures for social anxiety that capture a broader spectrum of symptoms may need to be developed, since the SIAS predominantly measures interaction anxiety and lacks components that assess performance anxiety and avoidance behaviors [86].

Some participants reported experiencing nausea in the VR conditions in our study, although not to the point where they asked to stop the experimental session. Other researchers have used physical movement input, linked to movement in the virtual world, to reduce the amount of nausea experienced in VR [87]. The addition of physical movement input may be beneficial in future work on the use of virtual forests in social anxiety therapy.

Future research should explore whether the observed trends in sentiment scores in this study persist across multiple therapy sessions and whether these emotional responses influence treatment adherence or therapy effectiveness. Additionally, examining how specific VR elements (such as interactivity, nature sounds, or dynamic visual changes) contribute to these effects could help refine VR therapy design for maximum benefit.

We used one virtual forest and two paths within that forest. Future research should examine how variations in the designed features of a virtual forest affect various aspects of user experience, including immersiveness, engagement, enjoyment, and susceptibility to therapeutic instruction and resulting benefits. Relevant features of the forest that could be systematically varied in such research include the number of interactive elements, visual and auditory stimuli, and the degree of participant movement within the VR environment.

## 6. Conclusions

Our study explored the usability and effectiveness of combining a virtual forest with therapeutic exercises to address social anxiety in young adults. Condition Non-VR showed a tendency to reduce social anxiety in contrast to the other conditions thereby answering our first research question in the negative (in the context of a short duration study), and our second research question in the positive), which suggests that a simple application that focuses on therapeutic content, without the distractions of a rich virtual environment, may be better at reducing social anxiety, at least in the short term. For our third research question, we didn’t find that adding therapeutic instruction lowered the user experience or positive sentiment associated with the virtual forest, but we did find that therapeutic instruction reduced immersiveness as measured by the number of immersive words and phrases used in open-ended comments. 

Condition B, combining virtual forest and therapeutic exercises, demonstrated benefits in enhancing long-term engagement and learning. Participants in this condition reported more positive experiences, and had greater quiz accuracy, indicating more accurate comprehension of therapy content. The virtual forest may sustain participant interest and engagement with therapeutic instruction, suggesting that the combined approach could be more effective in maintaining attention and fostering deeper comprehension of the therapy content over time. However, there may be potential for cognitive overload when therapeutic instructions are combined with an engaging immersive environment. It is possible that overload may have reduced the immersiveness of the virtual forest when therapy instructions were added (as shown by the large drop in Figure 7 in immersiveness for Condition B relative to the VR-only condition, i.e., condition A). Thus there is a need for careful design in therapeutic virtual forests to create appropriate levels of immersiveness and engagement, but without inducing cognitive load or compromising the effectiveness of therapy.

These findings highlight the importance of the delivery mode in therapeutic interventions. While the virtual forest offers an engaging and immersive experience that may enhance long-term learning and engagement, traditional non-immersive methods may facilitate a better immediate reduction in social anxiety. Future research should aim to optimize VR therapy by balancing the immersive elements of the virtual environment with features that help the participant focus on therapeutic content. One possibility may be to use simplified environments that are less immersive and distracting than VR while retaining some of the features and benefits of a forest bathing experience (e.g., a forest video, or succession of still images, on a large screen, combined with forest sounds and other ambient sound effects).

Virtual forest bathing is a promising environment for delivering therapeutic content. However, careful design is required to ensure the technology does not become distracting or interfere with the reception and understanding of therapeutic content. One key issue is how to deliver therapeutic content over time. For example, therapy could start in non-VR settings and progress to VR in later sessions to maintain participants’ interest and engagement in the therapeutic process. Another consideration is individual differences—some individuals may be particularly prone to cybersickness and may benefit from less immersive environments, such as images of forests combined with forest sounds.

Beyond general therapeutic settings, virtual forest technologies have notable practical applications. For example, these tools could be introduced to people with disabilities who are unable to physically visit a forest, elderly individuals who are homebound or live in nursing homes, leukemia patients undergoing treatment who have weakened immune systems and cannot interact with the external environment, or patients in closed institutions such as prisons undergoing rehabilitation. These populations, who may have limited access to natural environments, could greatly benefit from virtual nature exposure as a safe and effective means of promoting psychological well-being. While the challenges discussed in this paper are complex and considerable work will be required to develop virtual forest technologies for therapeutic interventions, the potential benefits of successfully implementing these technologies should far outweigh the research effort needed to develop them.

## Figures and Tables

**Table 1:** Number of participants in each condition and session.

Session	A	B	Non-VR
1	18	22	18
2		5	4

**Table 2:** Factor loadings of the SIAS 20 items.

SIAS Items	Factor Loadings
1. I get nervous if I have to speak with someone in authority (teacher, boss, etc.).	0.637
2. I have difficulty making eye contact with others.	0.509
3. I become tense if I have to talk about myself or my feelings.	0.472
4. I find it difficult to mix comfortably with the people I work with.	0.803
5. I find it difficult to make friends my own age.	0.359
6. I tense up if I meet an acquaintance in the street.	0.606
7. When mixing socially, I am uncomfortable.	0.768
8. I feel tense if I am alone with just one other person.	0.380
9. I have difficulty meeting people at parties, etc.	0.371
10. I have difficulty talking with other people.	0.707
11. I find it difficult to think of things to talk about.	0.433
12. I worry about expressing myself in case I appear awkward.	0.749
13. I find it difficult to disagree with another’s point of view.	0.529
14. I have difficulty talking to people I am attracted to.	0.184
15. I find myself worrying that I won’t know what to say in social situations.	0.722
16. I am nervous mixing with people I don’t know well.	0.692
17. I feel I’ll say something embarrassing when talking.	0.814
18. When mixing in a group, I find myself worrying I will be ignored.	0.601
19. I am tense mixing in a group.	0.771
20. I am unsure whether to greet someone I know only slightly.	0.539

Note. Items above are from the Social Interaction Anxiety Scale (SIAS) [56].

**Table 3:** Five-item scale retained after item reliability analysis.

Phase and Cronbach’s Alpha	SIAS Items
S1 Pre = 0.87 S1 Post = 0.93	7. When mixing socially, I am uncomfortable. 12. I worry about expressing myself in case I appear awkward. 15. I find myself worrying that I won’t know what to say in social situations. 17. I feel I’ll say something embarrassing when talking. 19. I am tense mixing in a group.

**Table 4:** Contingency Table of Participant Accuracy in the Three Checkpoints.

Checkpoint	Condition	Correct	Incorrect
1	B	41	1
	Non-VR	35	1
2	B	19	2
	Non-VR	17	1
3	B	40	2
	Non-VR	12	18

Note. These data were collected from a total of 21 participants in Condition B and 18 participants in Condition Non-VR; Checkpoints 1 and 3 have two questions. Data from one participant in Condition B across all three sessions and three participants in Condition Non-VR in Checkpoint 3 were lost due to technical issues.

**Table 5:** Top and bottom sentiment score comments.

Condition	Comments	Sentiment Score
Comments with highest three sentiment scores across three conditions		
A	I found the sounds and relaxing graphics to be of great stress relief. i also found that it was easy to become immersed into and feel a great sense of calm. The structures were nice as they were all different and invoked a sense of interest and wonder. I enjoyed the experience thoroughly.	0.98
B	I like how there are cute animals included in there, but it’ll be better if they are all pushable (pushing animals is satisfying idk why). Also, when I was exploring the river I was expecting something under the surface (maybe fish) but turns out there’s none, which makes me feel kind of disappointed… But overall the experience is good, the forest is pretty and the sound effect is really realistic. I’ll come back if I have time. Thank you for the experiment!	0.95
Non-VR	I enjoyed the modules, very helpful, thank you.	0.85
Comments with lowest three sentiment scores across three conditions		
A	I loved the environment that was created with the headset and I found myself immersed in the VR world but the feeling of nausea I had took me out of the experience and was the only thing that made me uncomfortable and what I didn’t like.	−0.33
B	I liked some of the images like the clouds, river, and flowers, but i also found it a bit distracting since i did feel i was in a virtual reality that was not real. It also made me nauseous, which made it harder to concentrate.	−0.04
Non-VR	Great experience but for module 4, I found it very boring and long so I may have lost focus there.	−0.64

**Table 6:** Participants’ comments on forest sounds.

Condition	Comments
A	“sound was very calming”
	“I really enjoyed the sounds and the graphics”
	“The sound and visual images made me feel like I am actually in the forest, so I felt calm.”
	“I feel like the visuals and the sounds were realistic enough to make my experience similar to a real-life experience.”
	“I think the environment that it built was really nice, but the soundtrack was the part that made it more alive.”
B	“An aspect that particularly stood out to me was the sound quality, which was really good (both the forest background noises and the checkpoint narrator’s voice).”
	“The aspect of the application that stood out to me was the sound quality. There was a lot of richness to the forest sounds, and the sound quality of the male narrator’s voice was also really good. It felt like it was coming from everywhere and nowhere at once.”
	“The forest is pretty and the sound effect is really realistic.”
Non-VR	“The background sounds were very relaxing and helped me visualize that I was in a forest.”
	“The sound effects were quite vivid which help immerse quickly.”

## Data Availability

In accordance with our ethics protocol, we would like to make our anonymized data openly accessible. This data includes demographics, questionnaire responses, and VR usage data, as well as the R and Python scripts used for data analysis. The data and scripts can be found on the Open Science Framework (OSF). Access to the data on OSF will be granted for a period of five years. Following this period, and in compliance with our commitment to destroy the data after five years, the data will be removed from OSF and deleted from our own database. OSF repository link: https://osf.io/se2mh/?view_only=e4a068740dd545adb2d1aec8cb589273 (accessed on 8 March 2025).

## Supplementary Materials

The virtual forest-only demonstration video can be downloaded at: https://drive.google.com/file/d/1drVrn3NyzZzrMKYAtaWJbKNTOqwD2rQv/view?usp=sharing (accessed on 8 March 2024); the combined virtual forest and therapeutic exercises demonstration video can be downloaded at: https://drive.google.com/file/d/1dqKkVuYRX-5_eCi42bPIxrcqFVnWRr-g/view?usp=sharing (accessed 8 March 2024).

## Appendix A. Psychotherapy Script

### Appendix A.1. CHECKPOINT #1: What Is Social Anxiety?

In accordance with a typical cognitive behavior therapy first session’s content, this checkpoint focused on psychoeducation [65]. The psychoeducation content was developed based on Andrews’ treatment manual (Pages 3–18). The psychoeducation part was followed by a quick abdominal breathing exercise (from Andrews’ manual) teaching people how to slow down the rate of respiration to cope with shallow and rapid breathing when anxiety level is high.

Anxiety is a normal part of life. Anxiety is part of an automatic response to threats that all animals share, known as the fight or flight response. It is designed to give the animal extra strength and speed in order to successfully flee from the threat, or, if trapped, to fight it. We often experience anxiety in anticipation of potentially troubling events, like an upcoming exam, an interview, a doctor’s appointment, or the focus of today’s activities. While there is a normal amount of nervousness to experience in social situations, it may be considered an anxiety disorder if the anxiety makes it difficult for you to function.

Do you constantly feel afraid that others are judging you? Does anxiety make it difficult for you to engage in everyday social situations, like at school, work, or a store?

If you are dealing with these feelings, you may have social anxiety, which is a fear of being scrutinized, evaluated, or the center of attention. However, the real underlying fear is of being evaluated negatively. Think of the reasons that you may believe you are being judged negatively:

- Will you say something that is embarrassing or awkward?
- Will others notice that you are anxious?
- Will you make a mistake?

You may worry that this negative evaluation will occur when interacting with others when you are doing something under observation or even in situations where there is a chance you will attract attention.

One of the common symptoms of the fight or flight response is the increase in the rate of respiration or overbreathing, which can contribute to feelings of anxiety. Breathing practices can be used as the foundation of your strategy to manage your anxiety, helping to calm you down so that you can think more clearly and apply the “straight thinking” strategies you will learn in the next few checkpoints. It can also be a useful strategy to shift your focus away from your anxious concerns and towards the breath and your body.

Meditation Exercise #1—Abdominal Breathing: We would like to teach you about abdominal breathing. Place one of your hands on your stomach if it is helpful. Placing your hand on your stomach may help you focus on the breath. Breathe in through the nose, pushing the belly outwards, and then breathe out with your mouth, feeling the belly move inwards. Focus on the feeling of the abdomen rising and falling with the in-breath and the out-breath. Non-judgmentally observing one’s own breathing from moment to moment, and bringing one’s attention back to the breath and the present moment when the mind wanders. To observe non-judgmentally, we try to not label our thoughts as right or wrong, we just accept them as they are. Breathing in slowly down into the belly and then exhaling through the mouth. To control our rate of respiration, we will now count to three as you inhale, and count to three again as you exhale. Take a deep breath in… one, two, three. Then a deep breath out… one, two, three. Now continue on your own for a few breaths, breathing in through the nose and out through the mouth and taking three seconds each for the in and out breaths. One last time, breathing in through the nose, filling the belly, expanding the lungs, and then letting everything go out through the mouth.

### Appendix A.2. CHECKPOINT #2: What Is Mindfulness?

This checkpoint introduced the concept of mindfulness, highlighting the concept of focusing on the present moment and becoming aware of thoughts and feelings based on Segal et al.’s mindfulness-based cognitive therapy manual [67]. To help cultivate awareness, participants were asked to engage in the four-senses mindfulness exercise (the five-sense exercise minus taste) to bring awareness to each sense [68].

To understand what mindfulness is, let us start with mindlessness. Often, we find ourselves doing things mindlessly, like driving a long distance in a car without being aware of the surroundings we are driving through. In a situation like a regular driving commute, we may end up driving on auto-pilot without really being aware of what is around us.

In contrast, mindfulness happens when we are fully aware of what we are sensing, doing, and thinking. It is as if we are committing to being in the place and situation where we are, rather than wishing to be somewhere else. Sometimes, being mindful is referred to as being present.

When we let our mind wander and do not practice mindfulness, we are at the whim of what thought passes into awareness. It has been said that the mind is like a naughty child that is always running off in different directions. And many of these directions may lead to negative thoughts that remind us of distressing or scary situations. If we want to control our state of well-being we need to control our state of mind.

Mindfulness is the awareness that emerges through paying attention. When we are mindful, we are living fully in the present moment. We are not thinking about the past or the future. We are not evaluating everything we perceive in terms of costs and benefits to us. We are not trying to grab something or run away. This concept of mindfulness is related to the idea of non-attachment, where we see things as they are without trying to attach ourselves to them in some way.

Mindfulness practices aim to help us to identify and become aware of our emotions and thoughts, and to avoid repetitive and negative patterns that arise from our fears and obsessions. Social anxiety is an enemy of mindfulness. Negative self-beliefs can be intrusive thoughts that the naughty child-like mind is constantly injecting into situations where this type of thought is not helpful and is probably not even relevant.

Mindfulness can teach us to foster self-compassion by treating these thoughts with curiosity and acceptance, rather than treating them with judgment and falling into repetitive thought patterns. When we love ourselves and feel compassion towards ourselves with respect to all the challenges we face, then it is easier to face situations in a constructive and mindful way. When we practice mindfulness, we are deliberately changing the focus of attention from our busy thoughts and speculations to the world that actually exists in front of, and around us.

Meditation Exercise #2—Four Senses Exercises: To begin with, we will bring our attention to each of our four senses to help ground ourselves in the present moment. We will also be training ourselves to put attention and awareness in different places at will.

1. Notice four things that you can see. Look around the forest and try to pick objects that you would not normally notice, like the vein of a leaf.
2. Notice three things that you can feel. Bring your attention to sensations that your body is feeling, like maybe the straps of the VR headset on your head.
3. Notice two things you can hear. Focus on two things that you can hear in the forest, maybe it is the bird chirping.
4. Notice one thing that you can smell. Take a moment to take note of one scent that you smell. Notice that normally, your attention is not focused on the scent, but mindfulness can help us return our attention to the scent when we wish.

This exercise helps us to turn our attention to the present moment. This is an exercise you can practice at any time if you find that your mind is not grounded in the here and now.

### Appendix A.3. CHECKPOINT #3: Challenging the Way You Think (Cognitive Restructuring)

We used both Andrews and Segal et al.’s manuals to develop this section of cognitive restructuring [65]. Challenging thoughts and cognitive restructuring are fundamental components of CBT. Participants also received two hypothetical situations, asking the participant to become aware of the thoughts that those situations evoke, to evaluate whether those thoughts are reasonable assessments and to reappraise the situation if the thoughts were not helpful.

Exercise #3—Part 1

Consider the following situation: You build up the courage to text one of your good friends, asking them to go out for lunch this weekend. They thank you for asking but tell you that they are busy this weekend.

Think about how you would feel in this situation. You may experience sadness, isolation, rejection, and resentment. Now think of the thoughts that would appear in your mind, and notice that the flavor of those thoughts is fueled by the emotions you would have felt:

- “They aren’t actually busy; they just do not want to hang out with me”
- “I built up the courage to ask, and the result is the same, I’m still lonely”
- “No one likes me”
- “I’m awkward, boring, weird, etc.”

When we experience emotions in situations, it is due to how we interpret those situations. In some situations, it is natural to feel stressed or experience emotions like fear because the situation is genuinely dangerous, just like being chased by a grizzly bear. But in most everyday situations, much of the stress and negative emotion that we experience is due to our interpretations of situations. And negative interpretations often happen because of bad habits of thinking that we have built up over time. For instance, think of how stress-inducing ordering food at a restaurant is for you. Notice that your level of stress is not universal; everyone experiences a different amount of stress when ordering food at a restaurant because it is our interpretation of the threat of the situation that causes the anxiety.

The exercises that we are asking you to do are designed to help you rethink how you see yourself and how you experience the world. We want you to re-examine the habits of thinking that are making you unhappy and anxious, whether it be ordering food or any other social situation. Identifying your existing habits of thinking is a necessary step towards creating new ways/habits of thinking, and new ways of interpreting situations that will make your life easier and happier.

Going back to the lunch example where your friend declined your offer, we will take a series of steps to re-evaluate the situation and create more helpful conclusions.

Step one: Become aware of the thoughts that populate in your mind, and accept them. These thoughts may be different for you, but for now, we will use the ones that we listed earlier.

Step two: Challenge the thoughts, deciding if they represent reasonable assessments of the situation. If you become critical of the first thought, such as “They aren’t actually busy, or they just do not want to hang out with me”, you may realize that it does not consider the possibility that they may have a packed weekend. Maybe their family is visiting, or maybe they are going on a trip, or maybe they have a fear of eating in public. Really, there are an infinite number of reasons that they said that, but the conclusion that your mind focuses on is the one that makes you feel bad about yourself.

Step three: Reappraise the situation. It may be worth giving yourself a moment to be proud of yourself for asking your friend to hang out—that may have required a lot of courage on your end. Acknowledge your effort and bravery in reaching out to your friend, regardless of their response. It’s essential to recognize that taking any step to connect with someone is a significant accomplishment, especially if you feel uncomfortable in social situations. Celebrate the fact that you initiated contact and that alone may have required a lot of courage on your end.

Next, you will try to challenge the “No one likes me” thought. Is this a reasonable thought? Why or why not?

If you do not know why the other person is unavailable in the example you just thought about, then it is unfair to interpret their response as evidence that they do not like you, and even more unfair to conclude that no one likes you. Even if this particular person does not want to hang out with you, it does not mean that there are no people out there who you can connect with and will value your presence. Sometimes, it can be difficult to find those people, but they are out there. Not everyone is compatible, as every individual has different values, interests, beliefs, etc. It is about finding the people who you share these things with.

Exercise #3—Part 2

To further illustrate the importance of being aware of your thoughts, consider the following example. Note your reaction to the two situations we are about to present:

Situation A: “You are feeling down because you’ve just had a quarrel with a colleague, Jim, at work. Shortly afterward, you see another colleague, Sarah, in the General Office, and they rush off quickly, saying they couldn’t stop to chat. What would you think?” [65]

Situation B: “You are feeling happy because you and a work colleague, Jim, have just been praised for good work. Shortly afterward, you see another colleague, Sarah, in the General Office, and they rush off quickly, saying they couldn’t stop to chat. What would you think?” [65]

In the first situation, someone may feel compelled to worry or to fall into other unproductive habitual behaviors. You may think of reasons why Sarah is also upset with you, or you might think that the entire office is working together against you. In the second situation, maybe you would see Sarah’s actions to be less about you. It is important to acknowledge that the experience with Jim, i.e., whether it is positive or negative, is going to affect the way that you interpret Sarah’s actions. By making this acknowledgement, you can respond more wisely to Sarah’s actions and perhaps appreciate that their actions may have been independent of your interaction with Jim.

Your feelings are always valid. We do not want you to reject the way that these uninvited thoughts make you feel; it is natural and valid to feel rejected, isolated, or sad if Jim or Sarah seemed upset with you. Rather, we want you to acknowledge and accept the way you feel, but also to be critical of the appraisals and interpretations you make in social situations, with the intention of restructuring those uninvited thoughts.

### Appendix A.4. CHECKPOINT #4: Mountain Meditation

The script below follows the mountain mediation developed by Kabat-Zinn [69].

This meditation is typically done seated, on the floor or in a chair. Start by connecting with the support beneath you, feeling the sensations of contact. Find a stable and poised posture, aligning your upper body over your hips and relaxing your shoulders.

Close your eyes and turn your attention to your breath. Feel each breath’s natural rhythm and flow.

Now, focus on a distant mountain. Gradually bring it into your awareness. Notice its shape, lofty peaks, solid base, and any distinctive features—snow-covered summits, trees, granite sides, or cascading streams.

As you observe the mountain, try to merge with it. Envision your body becoming the mountain, with your head as the peak and your body mirroring its form. Feel rooted, grounded, and unmoving, like the mountain.

With each breath, sense yourself becoming a breathing mountain, alive yet unwavering in your inner stillness.

As you sit here, becoming aware of the fact that as the sun travels across the sky, the light and shadows and colours are changing virtually moment by moment in the mountain’s stillness, and the surface teems with life and activity… streams, melting snow, waterfalls, plants and wildlife.

As the mountain sits, seeing and feeling how night follows day and day follows night. The bright warming sun, followed by the cool night sky studded with stars, and the gradual dawning of a new day…

Through it all, the mountain just sits, experiencing change in each moment, constantly changing, yet always just being itself. It remains still as the seasons flow into one another and as the weather changes moment by moment and day by day, calmness abiding all change…

In summer, there is no snow on the mountain except perhaps for the very peaks or in crags shielded from direct sunlight

In the fall, the mountain may wear a coat of brilliant fiery colours.

In winter, a blanket of snow and ice.

In any season, it may find itself at times enshrouded in clouds or fog or pelted by freezing rain.

People may come to see the mountain and comment on how beautiful it is or how it’s not a good day to see the mountain, that it’s too cloudy or rainy or foggy or dark.

None of this matters to the mountain, which remains at all times its essential self. Clouds may come and clouds may go, tourists may like it or not. The mountain’s magnificence and beauty are not changed one bit by whether people see it or not, seen or unseen, in sun or clouds, broiling or frigid, day or night.

It just sits, being itself.

At times visited by violent storms, buffeted by snow and rain and winds of unthinkable magnitude. Through it all, the mountain sits.

Spring comes, trees leaf out, flowers bloom in the high meadows and slopes, birds sing in the trees once again. Streams overflow with the waters of melting snow.

Through it all, the mountain continues to sit, unmoved by the weather, by what happens on its surface, by the world of appearances… remaining its essential self, through the seasons, the changing weather, the activity ebbing and flowing on its surface…

In the same way, as we sit in meditation, we can learn to experience the mountain, we can embody the same central, unwavering stillness and groundedness in the face of everything that changes in our own lives, over seconds, over hours, over years. In our lives and in our meditation practice, we constantly experience the changing nature of mind and body and of the outer world, we have our own periods of light and darkness, activity and inactivity, our moments of colour and our moments of drabness.

It’s true that we experience storms of varying intensity and violence in the outer world and in our own minds and bodies, buffeted by high winds, by cold and rain, we endure periods of darkness and pain, as well as the moments of joy and uplift, even our appearance changes constantly, experiencing a weather of it’s own…

By becoming the mountain in our meditation practice, we can embody its strength and stability and adopt them for our own. We can use its essence to support our energy to encounter each moment with mindfulness and equanimity and clarity.

It may help us to see that our thoughts and feelings, our preoccupations, our emotional storms and crises, and even the things that happen to us are very much like the weather on the mountain. We tend to take it all personally, but we can find wisdom in a mountain’s ability to remain stable amid the changing weather around it.

The weather of our own lives is not to be ignored or denied, it is to be encountered, honoured, felt, known for what it is, and held in awareness… And in holding it in this way, we come to know a deeper silence and stillness and wisdom.

Mountains have much to teach us. If you resonate with their strength, use them as a meditation anchor. Picture a mountain, and let it remind you to sit mindfully with resolve and wakefulness, in true stillness.

### Appendix A.5. CHECKPOINT #5: Avoidance and Aversion

Using Andrews’ manual (Pages 33–34), the material in this checkpoint discussed avoidance and its negative impact on social anxiety [65]. While avoidance might seem helpful, it often exacerbates anxiety and prevents personal growth.

Exercise #5—Part 1

In this section, we will talk about avoidance. If you recall the fight-or-flight-response, you may remember that we have a natural tendency to avoid situations that cause us distress, but if we do this with social situations, it can lead to the development or amplification of a social anxiety disorder. Imagine you are asked to go to a friend’s birthday party, where you only know a few of the 15 invitees. You may immediately think, “Oh no. There’s no way I could do that [65]. Some of them don’t want me there. What would I say to all those new people?”. You tell your friend that you will go, but when the day of the party rolls around, you become very anxious about attending the party.

Let’s say that your anxiety gets the best of you, and you come up with some excuse as to why you can no longer attend. You then feel much calmer. You may be thinking, “Phew! That was a lucky escape. I would have made a fool of myself” [65].

However, you probably also feel disappointed in yourself and perhaps even quite critical of yourself for not attending the party [65]. You might see pictures of your friends celebrating the party, and this makes you feel ashamed. You also lose the opportunity to discover that maybe you would have had a good time at the party, maybe even meeting some new people. Note, once again, the importance of your appraisal/interpretation, and how it is shaping your behavior and emotions. Your mind generated a thought, telling you that you were going to look silly if you attended the party, and you took that thought as truth.

In this situation, you used avoidance to deal with your anxieties. Acknowledge that your mind chooses avoidance because it appears to be helpful for you; that is you don’t have to risk the chance that you are embarrassed, you feel awkward, weird, etc., when attending the party. It is also important to acknowledge that there are several important consequences of avoidance [65]:

First, the original fear is strengthened or reinforced. That is, you strongly believe that had you not avoided the situation you would surely have embarrassed yourself because of your incompetence [65]. It makes it even harder to attend the next party, because your mind has learned to fear that situation even more. “If I do not find an excuse, I am sure to embarrass myself!”.

Second, you miss the opportunity to have an experience that might disprove or reduce your negative beliefs about yourself and start to build some confidence [65].

Third, you miss the opportunity to practice your social skills: most people only develop confidence in their social skills because of frequent practice, including learning from their mistakes [65].

Exercise 5 & Question—Part 2

There are plenty of instances where avoidance may appear as a useful tactic to deal with your social anxiety. Maybe you avoided a social gathering, or maybe you did not approach your boss when you were mistreated at work. Even in the party situation, you may sometimes have good reasons to avoid going. For example, you may want to avoid meeting someone you previously had a toxic relationship with.

The other major problem with using avoidance to try to control anxiety is that fears often seem to spread or generalize across situations. For example, you might start out being anxious about speaking on the telephone only when someone who makes you nervous is close enough to overhear. You might then try to avoid that situation, but gradually you find that you’re not really comfortable using the phone when anyone is nearby… with time, you find that you worry a lot about what the person on the other end might be thinking about what you have to say, too… eventually you find yourself trying to avoid all phone conversations… you don’t make calls and you won’t answer the phone… [65]

In the situation with your friend’s birthday party, we can generalize by saying that you experienced an unpleasant emotion, namely the fear of feeling awkward. Unpleasant emotions are a part of life, everyone experiences them. When you experience unpleasant emotions, like sadness, anger, embarrassment, or anxiety, how do you typically respond? For many, the answer is that they try to get rid of those feelings.

Mindfulness approaches are not about thought control or substituting positive for negative images of the past, present, or future. Rather, they offer ways for people to allow these feelings of disappointment and regret to be here. Our cell phones, TVs, laptops, etc., afford us the opportunity to distract ourselves from our painful experiences, instead of accepting them. On the other hand, when we worry or ruminate about our problems, although we may seem to be addressing the difficulty, such rumination actually takes us further away from a direct sense of calm, we are in a mode where we are thinking about the feelings rather than directly experiencing them [65].

### Appendix A.6. CHECKPOINT #6: Sounds Meditation

The sixth checkpoint exercise was sound meditation, adapted from Segal et al. to engage participants with forest sound played in the VR headset [67].

In the previous sessions, we had worked on shifting our awareness to sensations from the five senses and now we will work on shifting our sensations strictly to hearing.

To settle ourselves, let’s take one big grounding deep breath in, and a big exhale through the mouth.

Bring your attention to the ears and then allow the awareness to open and expand, so that there is a receptiveness to sounds as they arise, wherever they arise [67].

There is no need to go searching for sounds or listening for particular sounds. Instead, as best you can, simply open your awareness so that it is receptive to sounds from all directions as they arise—sounds that are close, sounds that are far away, sounds that are in front, behind, to the side, above, or below. Open to a whole space of sound around you. Be aware of obvious sounds and of more subtle sounds, aware of the space between sounds, and aware of silence [67].

Whenever you notice that your awareness is no longer focused on sounds at the moment, gently acknowledge where the mind had moved to, and then retune the awareness back to sounds as they arise and pass from one moment to the next [67].

And when you are ready, bring the sitting to a close by returning to the breath for one last inhale, and an exhale through the mouth [67].

As this practice comes to a close, reflect upon how you are feeling now… notice that when your mind is focused on the sounds, you are free from all your worries. Notice that you may be feeling refreshed, relaxed and/or rejuvenated. Thank yourself for taking the time to help yourself feel better.

### Appendix A.7. Self-Compassion

The seventh checkpoint exercise was about self-compassion, which incorporates Hofmann et al.’s Loving-kindness Meditation [70]. It discusses the inevitability of setbacks in conquering social anxiety and emphasizes the importance of treating oneself kindly when encountering those difficult times.

Think of all the New Year’s resolutions people make. Why do they often fail? Let’s say someone set the goal to go to the gym every day in the upcoming year. They are successful for the first 5 days, but then they can’t get themselves to go for the 6th day. Instead of focusing on the success of going to the gym for 5 days, they fixate on their first failure, and appraise the situation as a loss. They beat themselves up, and they begin to think of themselves as a failure. However, they may have set themselves up for failure; asking yourself to go to the gym every single day, without any relapse, is very difficult to do.

Similarly, if your social-anxiety-related goals ask for perfection, then you may have a hard time achieving those goals, and that failure can spiral into negative self-talk. Often, people will abandon their goals and aspirations when they face a setback. However, setbacks are inevitable, and they are absolutely part of the process of change. Setbacks can be a learning experience and result in greater confidence [70].

Many sufferers of social anxiety have low self-esteem. They are highly critical of themselves when they fail to meet their own expectations. It is essential to learn how to give yourself support and encouragement—it means more when it comes from within. Be kind to yourself when you don’t do as well as you’d have liked—how would you respond to someone else, or, especially, a child when they “fail”? Congratulate yourself on your effort as well as your successes. Consider setting yourself small rewards for meeting goals. Set your goals at realistic levels and learn to appreciate small successes. It may be only a small step, but if you keep taking small steps you will eventually reach your goals. Mastering anxiety is going to take time. It is challenging and tiring and difficult, and no one else really knows what it’s like. Learn to be kind to yourself, and you’ll not only endure but you’ll find it easier to question how much others’ opinions truly affect you [70].

Be kind to yourself, and you’ll not only endure but also find it easier to [70].

Don’t panic when you face setbacks, but go back to basics—revise what you have learned today. Seek help from others if you need it. Learn to be kind to yourself.

Meditation Exercise #7—Loving-kindness Meditation

The loving-kindness meditation that we will now practice can help us recognize qualities of warmth and compassion, towards yourself and others.

Whenever you are ready, bring your awareness to the breathing and the body as a whole. Resting here for a period of time, establishing a relatively stable platform of moment-to-moment awareness, riding on the waves of the breath [70].

When you feel comfortable resting within the natural flow of your breathing, picturing in your mind’s eye someone in your life who loves you or loved you totally and unconditionally [70].

Recall the feeling of love and kindness they accorded you, and the whole aura of their love for you. Breathing with these feelings, bathing in them, resting in their total acceptance and embracing of you just as you are or were. Acknowledge that you are loved and accepted without having to be different, without having to be worthy of their love, without having to be particularly deserving. In fact, you may not feel particularly worthy or deserving. That does not matter. In fact, it is irrelevant. The relevant fact is that you were or are loved wholeheartedly. Their love is or was for you just as you are, for who you are now, already, and perhaps always have been. It is truly unconditional [70].

Now, whenever you feel ready, see if you can become the source as well as the receiver of these same feelings—in other words, take on these very same loving feelings for yourself as if they were your own rather than those of another. Linger with the rhythmic beating of your own heart, inviting into your own heart these feelings of love and acceptance and kindness for yourself, beyond the judgment of any kind, just basking in feelings of loving-kindness akin to the embrace of a mother for her child, where you are simultaneously both the mother and the child. Resting here in these feelings as best you can from moment to moment, breath by breath, bathing in your own kind regard and acceptance of yourself as you are. Letting this feeling be self-sustaining, natural, in no way forced or coerced. Even tiny tastes of it can help to relieve you from all all the negativity and self-criticism, and self-loathing that can lie beneath the surface of our psyches [70].

In resting here in this field of loving-kindness, this embrace of loving-kindness, you may find it useful to whisper to yourself inwardly the following phrases or hear them being whispered to you by the wind, by the air, by the breath, by the world [70]:

May I be safe and protected and free from inner and outer harm

May I be happy and contented.

May I be healthy and whole to whatever degree possible.

May I experience ease of well-being…

Now, as the loving-kindness practice comes to a close, thank yourself for participating. We who choose to practice and embody loving-kindness, formally or informally, if even just a little bit, are undisputedly its first but hardly its only beneficiaries [70].

### Appendix A.8. CHECKPOINT #8: Closure & Review

The eighth checkpoint was similar to a closure session, which guides participants to reflect on what they have learned and how they can apply it in the real world, followed by a simple breathing meditation, Three Minute Breathing Space [66,67].

We deeply appreciate your active participation until the final session! We started off with the introduction of social anxiety, and discussed abdomen breathing as a breathing technique that can help us manage anxiety. Mindfulness was then introduced as a practice that can help us abandon our “autopilot” mode, and instead become aware of our relapse-related automatic thought patterns when they are triggered. We then learned about skillful ways of responding to those thoughts, like in the lunch-with-a-friend example. We do not deny that these automatic, negative thoughts exist, rather we choose to hold those thoughts and feelings into a more spacious awareness, anchored in the present moment by the breath [66,67]. Furthermore, an unproductive way of responding to unpleasant thoughts is avoidance, as avoiding the situations we fear tightens the grip that they have on our lives. We allow ourselves to see the unpleasant feelings clearly, to explore the difficulty itself and our reactions to it with gentleness and compassion [66,67]. We practiced awareness of bodily sensations and sounds, and finished with awareness of our thoughts, working to appreciate the temporary nature of these events. Finally, we talked about the importance of being kind to yourself, as it will be paramount to treat yourself with compassion when you face setbacks in your journey to tackle social anxiety.

Meditation Exercise #8—Three Minute Breathing Space [66,67]

To come full circle, we will finish with a final 3-min meditative practice that can help us step out of automatic pilot, into the present moment and slow down so you can respond more skillfully to stressful situations.

Step 1:

Notice what is going on with you right now

Whatever thoughts may be around

Whatever feelings or emotions…any sensations in the body

Just tuning into what the weather pattern inside you is right now

Continue being open to this for a few moments

As best you can let go of the tendency that we all have to want things to be different than they are right now.

See if you can allow things to be just as they are right now in your mind and in your body.

Step 2:

Gathering your attention on your breath…down in your abdomen as you breathe in and out. Not trying to make the breath do anything special or be different than how you found it…just focusing on the sensations of the in-breath and the out-breath.

And if the mind wanders, gently escorting the attention back to the breath and the sensations of breathing.

Step 3:

Expand attention to the body of the whole…as if the whole body could breathe right now. You could be aware of all the sensations in the body from the top of the head to the toes to the fingertips…to the sensations of the skin…and all the sensations inside your body as well. Aware of the whole landscape of sensations in the body.

Once again, see if you can allow the sensations to be exactly as find them…not trying to change them in any way. A sense of opening to what’s here right now.

A sense of coming home to the body and allowing yourself to be exactly as you are…moment by moment…breath by breath.

And then, at a certain point, begin to move your fingers and toes…letting your eyes open if they were closed…and resuming the activities of your day. This 3-min breathing space provides a way to step out of automatic pilot and reconnect with the present moment.

## Appendix B. Word List for Occurrences and Theme Analysis

“Immersiveness”, “immersed”, “immersive”, “immersing”, “immerse”, “immersion”, “realistic”, “being in nature”, “helped me visualize”, “took me out of the real world”, “made me feel like I am actually in the forest”, “being in the virtual environment”, “similar to a real-life experience”.
